# Supplementary material for: Positive Psychology Approaches to Interventions for Cancer Dyads: A Scoping Review
Source: Int J Environ Res Public Health. 2022 Oct 19;19(20):13561. doi: 10.3390/ijerph192013561 (PMC9602591; doi:10.3390/ijerph192013561)
Supplement: Supplementary file 1 [file ijerph-19-13561-s001.zip › ijerph-1949183-supplementary.pdf]

International Journal of
Environmental Research and Public Health

MDPI

Supplementary Materials

Positive Psychology Approaches to Interventions for Cancer Dyads: A Scoping Review

Supplemental Table S1. Search Keywords & PICOS Question.

**Positive Psychology Constructs**	**Positive Psychology Interventions**	**Caregiver Terms**	**Cancer Terms**
Benefit finding	hope therapy	caregiv*	Cancer*
blessing	mindfulness	care giver*	Oncology
dispositional optimism	quality of life therapy	carer*	Neoplasms[Mesh]
engagement	strength-centered therapy	family	
flow	well-being therapy	famil*	
gratitude	″psychotherapeutic interventions″	spouse*	
happiness	″psychological interventions″ [tiab]	relative*	
hardiness	″psychotherapy″ [tiab]	partner*	
hope	″psychotherapy interventions″ [tiab]	Caregivers[Mesh]	
humor	″behavioral therapy″ [tiab]	Spouses[Mesh]	
kindness	″behaviour therapy″ [tiab]	Family Therapy[Mesh]	
meaning*	″behavioural therapy″ [tiab]		
meaning-making	″positive psychotherapy″ [tiab]		
optimism	″positive psychology″ [tiab]		
personal growth	positive psycho* [tiab]		
personal strength	″psychological intervention″ [tiab]		
positive affect	″therapeutic intervention″ [tiab]		
positive coping	″social constructionist″ [tiab]		
positive emotions	″problem solving therapy″ [tiab]		
positive psychological growth	″supportive therapy″ [tiab]		
positive reappraisal coping	″meaning centered psychotherapy″ [tiab]		
positive relationships	″positive emotion communication″ [tiab]		
positivity	″psychoeducational group interventions″ [tiab]		
posttraumatic growth	″psychoeducational interventions″ [tiab]		
resilience	″behavioral interventions″ [tiab]		
satisfaction	″behavioural interventions″ [tiab]		
savoring	Psychosocial intervention* [tiab]		
spirituality	psychosocial [tiab]		
well-being	"Psychosocial Support Systems"[Mesh]		
"Mindfulness"[Mesh]	"Psycho-Oncology"[Mesh]		
"Hope"[Mesh]			
Posttraumatic Growth, Psychological[Mesh]			
Optimism[Mesh]			

Adaptation,<br>Psychological[Mesh]<br>Virtue<br>Forgiveness<br>Flourish<br>Savor*<br>Empower*			
**PICO** (P = participants/population, I = Interventions/exposures, C = comparators/controls, and O = outcomes; primary and secondary outcomes, S= study type)<br><br>**P** = Cancer patients/survivors and their primary support person (e.g., spouse/partner, family caregiver)<br>**I** = Positive psychology interventions<br>**C** = N/A<br>**O** = N/A<br>**S** = Original data, observational data, (not a review, meta-analysis, secondary data analysis, case report, case series, commentary, RETROSPECTIVE, REGISTRY STUDIES, etc...). Clinical Trials, Randomized Control Trials, etc. Expanded access trials may be included.			

Supplemental Table S2. Search Strings by Database.

**PubMed.gov Search**	
**#**	**PubMed Keywords & Translations**
1	((("Benefit finding" [tiab] OR blessing* [tiab] OR "dispositional optimism" [tiab] OR engagement [tiab] OR gratitude* [tiab] OR happiness [tiab] OR hardiness [tiab] OR hope [tiab] OR humor [tiab] OR kindness [tiab] OR meaning* [tiab] OR "meaning-making" [tiab] OR optimism [tiab] OR "personal growth" [tiab] OR "personal strength" [tiab] OR "positive affect" [tiab] OR "positive coping" [tiab] OR "positive emotions" [tiab] OR "positive psychological growth" [tiab] OR "positive reappraisal coping" [tiab] OR "positive relationships" [tiab] OR positivity [tiab] OR "posttraumatic growth" [tiab] OR resilience [tiab] OR satisfaction [tiab] OR savoring [tiab] OR spirituality [tiab] OR "well-being" [tiab] OR Quality of Life[Mesh] OR "Mindfulness"[Mesh] OR "Hope"[Mesh] OR Posttraumatic Growth, Psychological[Mesh] OR Optimism[Mesh] OR Adaptation, Psychological[Mesh] OR Virtue* [tiab] OR Forgiveness [tiab] OR Flourish [tiab] OR Savor* [tiab] OR Empower* [tiab] OR Humor [tiab] OR "Positive psychological functioning" [tiab])))
2	((″psychotherapeutic interventions″ OR ″psychological interventions″ [tiab] OR ″psychotherapy″ [tiab] OR ″psychotherapy interventions″ [tiab] OR ″behavioral therapy″ [tiab] OR ″behaviour therapy″ [tiab] OR ″behavioural therapy″ [tiab] OR ″positive psychotherapy″ [tiab] OR ″positive psychology″ [tiab] OR positive psycho* [tiab] OR ″psychological intervention″ [tiab] OR ″therapeutic intervention″ [tiab] OR ″social constructionist″ [tiab] OR ″problem solving therapy″ [tiab] OR ″supportive therapy″ [tiab] OR ″meaning centered psychotherapy″ [tiab] OR ″positive emotion communication″ [tiab] OR ″psychoeducational group interventions″ [tiab] OR ″psychoeducational interventions″ [tiab] OR ″behavioral interventions″ [tiab] OR ″behavioural interventions″ [tiab] OR Psychosocial intervention* [tiab] OR psychosocial [tiab] OR "Psychosocial Support Systems"[Mesh] OR "Psycho-Oncology"[Mesh]))
3	((((("Benefit finding" [tiab] OR blessing* [tiab] OR "dispositional optimism" [tiab] OR engagement [tiab] OR gratitude* [tiab] OR happiness [tiab] OR hardiness [tiab] OR hope [tiab] OR humor [tiab] OR kindness [tiab] OR meaning* [tiab] OR "meaning-making" [tiab] OR optimism [tiab] OR "personal growth" [tiab] OR "personal strength" [tiab] OR "positive affect" [tiab] OR "positive coping" [tiab] OR "positive emotions" [tiab] OR "positive psychological growth" [tiab] OR "positive reappraisal coping" [tiab] OR "positive relationships" [tiab] OR positivity [tiab] OR "posttraumatic growth" [tiab] OR resilience [tiab] OR satisfaction [tiab] OR savoring [tiab] OR spirituality [tiab] OR "well-being" [tiab] OR Quality of Life[Mesh] OR "Mindfulness"[Mesh] OR "Hope"[Mesh] OR Posttraumatic Growth, Psychological[Mesh] OR Optimism[Mesh] OR Adaptation, Psychological[Mesh] OR Virtue* [tiab] OR Forgiveness [tiab] OR Flourish [tiab] OR Savor* [tiab] OR Empower* [tiab] OR

	Humor [tiab] OR "Positive psychological functioning" [tiab]))))) AND (″psychotherapeutic interventions″ OR ″psychological interventions″ [tiab] OR ″psychotherapy″ [tiab] OR ″psychotherapy interventions″ [tiab] OR ″behavioral therapy″ [tiab] OR ″behaviour therapy″ [tiab] OR ″behavioural therapy″ [tiab] OR ″positive psychotherapy″ [tiab] OR ″positive psychology″ [tiab] OR positive psycho* [tiab] OR ″psychological intervention″ [tiab] OR ″therapeutic intervention″ [tiab] OR ″social constructionist″ [tiab] OR ″problem solving therapy″ [tiab] OR ″supportive therapy″ [tiab] OR ″meaning centered psychotherapy″ [tiab] OR ″positive emotion communication″ [tiab] OR ″psychoeducational group interventions″ [tiab] OR ″psychoeducational interventions″ [tiab] OR ″behavioral interventions″ [tiab] OR ″behavioural interventions″ [tiab] OR Psychosocial intervention* [tiab] OR psychosocial [tiab] OR "Psychosocial Support Systems"[Mesh] OR "Psycho-Oncology"[Mesh])
4	(caregiv* [tiab] OR care giver* [tiab] OR carer* [tiab] OR family [tiab] OR famil* [tiab] OR spouse* [tiab] OR relative* [tiab] OR partner* [tiab] OR Caregivers[Mesh] OR Spouses[Mesh] OR Family Therapy[Mesh])
5	(Cancer* [tiab] OR Oncology [tiab] OR Neoplasms[Mesh])
6	(mice [tiab] OR mouse [tiab] OR tumor metabolism [tiab] OR node [tiab] OR nodal [tiab] OR circulating tumor cells [tiab] OR molecule [tiab] OR molecular [tiab] OR phenotype [tiab] OR biopsy [tiab] OR hormone receptor [tiab] OR tissue [tiab] OR cell [tiab] OR benign [tiab] OR antibod* [tiab] OR gene [tiab] OR genetic [tiab] OR receptor* [tiab] OR tumor microenviroment [tiab] OR placebo [tiab] OR ncRNAs [tiab])
7	((((((((("Benefit finding" [tiab] OR blessing* [tiab] OR "dispositional optimism" [tiab] OR engagement [tiab] OR gratitude* [tiab] OR happiness [tiab] OR hardiness [tiab] OR hope [tiab] OR humor [tiab] OR kindness [tiab] OR meaning* [tiab] OR "meaning-making" [tiab] OR optimism [tiab] OR "personal growth" [tiab] OR "personal strength" [tiab] OR "positive affect" [tiab] OR "positive coping" [tiab] OR "positive emotions" [tiab] OR "positive psychological growth" [tiab] OR "positive reappraisal coping" [tiab] OR "positive relationships" [tiab] OR positivity [tiab] OR "posttraumatic growth" [tiab] OR resilience [tiab] OR satisfaction [tiab] OR savoring [tiab] OR spirituality [tiab] OR "well-being" [tiab] OR Quality of Life[Mesh] OR "Mindfulness"[Mesh] OR "Hope"[Mesh] OR Posttraumatic Growth, Psychological[Mesh] OR Optimism[Mesh] OR Adaptation, Psychological[Mesh] OR Virtue* [tiab] OR Forgiveness [tiab] OR Flourish [tiab] OR Savor* [tiab] OR Empower* [tiab] OR Humor [tiab] OR "Positive psychological functioning" [tiab]))))) AND (″psychotherapeutic interventions″ OR ″psychological interventions″ [tiab] OR ″psychotherapy″ [tiab] OR ″psychotherapy interventions″ [tiab] OR ″behavioral therapy″ [tiab] OR ″behaviour therapy″ [tiab] OR ″behavioural therapy″ [tiab] OR ″positive psychotherapy″ [tiab] OR ″positive psychology″ [tiab] OR positive psycho* [tiab] OR ″psychological intervention″ [tiab] OR ″therapeutic intervention″ [tiab] OR ″social constructionist″ [tiab] OR ″problem solving therapy″ [tiab] OR ″supportive therapy″ [tiab] OR ″meaning centered psychotherapy″ [tiab] OR ″positive emotion communication″ [tiab] OR ″psychoeducational group interventions″ [tiab] OR ″psychoeducational interventions″ [tiab] OR ″behavioral interventions″ [tiab] OR ″behavioural interventions″ [tiab] OR Psychosocial intervention* [tiab] OR psychosocial [tiab] OR "Psychosocial Support Systems"[Mesh] OR "Psycho-Oncology"[Mesh]))) AND ((Cancer* [tiab] OR Oncology [tiab] OR Neoplasms[Mesh])))) AND ((caregiv* [tiab] OR care giver* [tiab] OR carer* [tiab] OR family [tiab] OR famil* [tiab] OR spouse* [tiab] OR relative* [tiab] OR partner* [tiab] OR Caregivers[Mesh] OR Spouses[Mesh] OR Family Therapy[Mesh]))
8	((((((((((("Benefit finding" [tiab] OR blessing* [tiab] OR "dispositional optimism" [tiab] OR engagement [tiab] OR gratitude* [tiab] OR happiness [tiab] OR hardiness [tiab] OR hope [tiab] OR humor [tiab] OR kindness [tiab] OR meaning* [tiab] OR "meaning-making" [tiab] OR optimism [tiab] OR "personal growth" [tiab] OR "personal strength" [tiab] OR "positive affect" [tiab] OR "positive coping" [tiab] OR "positive emotions" [tiab] OR "positive psychological growth" [tiab] OR "positive reappraisal coping" [tiab] OR "positive relationships" [tiab] OR positivity [tiab] OR "posttraumatic growth" [tiab] OR resilience [tiab] OR satisfaction [tiab] OR savoring [tiab] OR spirituality [tiab] OR "well-being" [tiab] OR Quality of Life[Mesh] OR "Mindfulness"[Mesh] OR "Hope"[Mesh] OR Posttraumatic Growth, Psychological[Mesh] OR Optimism[Mesh] OR Adaptation, Psychological[Mesh] OR Virtue* [tiab] OR Forgiveness [tiab] OR Flourish [tiab] OR Savor* [tiab] OR Empower* [tiab] OR Humor [tiab] OR "Positive psychological functioning" [tiab]))))) AND (″psychotherapeutic interventions″ OR ″psychological interventions″ [tiab] OR ″psychotherapy″ [tiab] OR ″psychotherapy interventions″ [tiab] OR ″behavioral therapy″ [tiab] OR ″behaviour therapy″ [tiab] OR ″behavioural therapy″ [tiab] OR ″positive psychotherapy″ [tiab] OR ″positive psychology″ [tiab] OR

	positive psycho* [tiab] OR ″psychological intervention″ [tiab] OR ″therapeutic intervention″ [tiab] OR ″social constructionist″ [tiab] OR ″problem solving therapy″ [tiab] OR ″supportive therapy″ [tiab] OR ″meaning centered psychotherapy″ [tiab] OR ″positive emotion communication″ [tiab] OR ″psychoeducational group interventions″ [tiab] OR ″psychoeducational interventions″ [tiab] OR ″behavioral interventions″ [tiab] OR ″behavioural interventions″ [tiab] OR Psychosocial intervention* [tiab] OR psychosocial [tiab] OR "Psychosocial Support Systems"[Mesh] OR "Psycho-Oncology"[Mesh]))) AND ((Cancer* [tiab] OR Oncology [tiab] OR Neoplasms[Mesh])))) AND ((caregiv* [tiab] OR care giver* [tiab] OR carer* [tiab] OR family [tiab] OR famil* [tiab] OR spouse* [tiab] OR relative* [tiab] OR partner* [tiab] OR Caregivers[Mesh] OR Spouses[Mesh] OR Family Therapy[Mesh]))))) NOT (((mice [tiab] OR mouse [tiab] OR tumor metabolism [tiab] OR node [tiab] OR nodal [tiab] OR circulating tumor cells [tiab] OR molecule [tiab] OR molecular [tiab] OR phenotype [tiab] OR biopsy [tiab] OR hormone receptor [tiab] OR tissue [tiab] OR cell [tiab] OR benign [tiab] OR antibod* [tiab] OR gene [tiab] OR genetic [tiab] OR receptor* [tiab] OR tumor microenviroment [tiab] OR placebo [tiab] OR ncRNAs [tiab])))
9	((("Benefit finding" [tiab] OR blessing* [tiab] OR "dispositional optimism" [tiab] OR engagement [tiab] OR gratitude* [tiab] OR happiness [tiab] OR hardiness [tiab] OR hope [tiab] OR humor [tiab] OR kindness [tiab] OR meaning* [tiab] OR "meaning-making" [tiab] OR optimism [tiab] OR "personal growth" [tiab] OR "personal strength" [tiab] OR "positive affect" [tiab] OR "positive coping" [tiab] OR "positive emotions" [tiab] OR "positive psychological growth" [tiab] OR "positive reappraisal coping" [tiab] OR "positive relationships" [tiab] OR positivity [tiab] OR "posttraumatic growth" [tiab] OR resilience [tiab] OR satisfaction [tiab] OR savoring [tiab] OR spirituality [tiab] OR "well-being" [tiab] OR Quality of Life[Mesh] OR "Mindfulness"[Mesh] OR "Hope"[Mesh] OR Posttraumatic Growth, Psychological[Mesh] OR Optimism[Mesh] OR Adaptation, Psychological[Mesh] OR Virtue* [tiab] OR Forgiveness [tiab] OR Flourish [tiab] OR Savor* [tiab] OR Empower* [tiab] OR Humor [tiab] OR "Positive psychological functioning" [tiab])))
**Final Action:** Export results for 8 to EndNote.	
**Cochrane Library Search**	
#	**Cochrane Search String**
1	caregiv* OR (care AND giver*) OR carer*
2	TS=(pembrolizumab OR keytruda)
3	(positive AND psychology) OR mindfulness OR (quality NEAR/1 life) OR well-being OR wellbeing OR (behavioral AND therapy) OR (behaviour AND therapy) OR psycho-oncology
4	#1 AND #2
5	MeSH descriptor: [Neoplasms] explode all trees
6	#3 AND #4
**Final Action:** Export results to EndNote & deduplicate	
**Embase Search**	
#	**Embase Search String**
1	('benefit-finding'/exp OR 'benefit-finding' OR blessing OR 'dispositional optimism' OR 'engagement' OR 'gratitude' OR 'happiness' OR 'hardiness' OR 'hope' OR humor OR 'kindness' OR 'meaning-making' OR 'optimism' OR 'personal growth' OR 'personal strength' OR 'positive affect' OR 'positive coping' OR 'positive emotions' OR 'positive psychological growth' OR 'positive reappraisal coping' OR 'positive relationships' OR 'positivity' OR 'posttraumatic growth' OR 'psychological resilience' OR 'satisfaction' OR savoring OR 'religion' OR 'wellbeing' OR 'mindfulness' OR 'posttraumatic growth (psychology)' OR 'coping behavior' OR 'morality' OR 'empowerment') AND ('caregiver' OR caregiv* OR 'family' OR famil* OR 'spouse' OR 'partner') AND ('malignant neoplasm'/exp OR 'malignant neoplasm') AND ('hope therapy' OR 'meaningful life therapy' OR 'mindfulness based therapy' OR 'mindfulness based stress reduction' OR 'mindfulness based cognitive therapy' OR 'mindfulness meditation' OR 'positive psychotherapy' OR 'positive psychology' OR 'positive psychology intervention' OR 'psychotherapy' OR 'social constructionism') AND [embase]/lim NOT ([embase]/lim AND [medline]/lim)<br>Mapped termsn/a
**Final Action:** Export results to EndNote.	

Supplemental Table S3. Study Inclusion & Exclusion Criteria.

**Inclusion Criteria**	**Exclusion Criteria**
1. Must be **human subjects research**<br>2. Must be **about cancer**<br>3. Must include an intervention **delivered to dyads**<br>a. Dyad = 1 patient/survivor + 1 support person (e.g., spouse/partner, caregiver, other family member, friend)<br>b. The support person is required to have a relationship with the patient prior to participation in the intervention (i.e., not assigned to the patient as part of the intervention).<br>c. Both members of the dyad have to be enrolled/participate in the intervention, but there does not need to be outcomes assessed/reported for both members of the dyad.<br>d. The intervention can be primarily focused on patients and include support people for only some of the intervention sessions, but it must include a support person for *all* of the enrolled patients (i.e., the intervention cannot include support people for only some of the enrolled patients, regardless of whether the support people that are enrolled participate in all sessions of the intervention).<br>e. The intervention must be delivered to both members of the dyad *together* for *at least part* of the intervention (i.e., a patient and support person receiving separate intervention sessions with no sessions together would not count as dyadic).<br>f. Group-based interventions may count as dyadic if the group consists of multiple dyads.<br>4. Must deal with **adult populations**<br>5. Must **contain an intervention with aspects of a positive psychology intervention**<br>a. Positive psychology intervention is the clinical or therapeutic arm of positive psychology. It integrates symptoms with strengths, risks with resources, weaknesses with values, and regrets with hopes, in order to understand the inherent complexities of human experience in a balanced way. It does not minimize or dismiss a client's concern but is considered a reorientation—"build what's strong" to supplement the traditional "fix what's wrong" approach; clients are taught to use their greatest resources—both personal and interpersonal—to meet life's challenges and actively facilitate growth, resilience, and well-being.<br>6. Article must be written in **English**<br>7. Article must be **peer-reviewed** (e.g., not an unpublished dissertation, book chapter, etc.)<br>8. Must be **original research** (e.g., not meta analysis or review)<br>9. Must **not be a case study**<br>10. Must **assess impact of intervention(s) on patient and/or spouse/caregiver/family member outcome(s)** (e.g., not a study protocol with no results reported, description of development/adaptation of intervention, or evaluation of impact of intervention on other types of processes or outcomes)	1. Is not human subjects research<br>2. Is not about cancer<br>3. Does not include an intervention delivered to dyads (e.g., only focused on patients/survivors, only focused on caregivers, etc.)<br>a. See notes under Inclusion Criterion #3 for details<br>4. Deals with children/pediatric populations<br>5. Does not contain an intervention with aspects of a positive psychology intervention<br>a. See note under Inclusion Criterion #5 for details<br>6. Article not written in English<br>7. Article not peer-reviewed (e.g., unpublished dissertation, book chapter, etc.)<br>8. Not original research (e.g., meta-analysis or review)<br>9. Case study<br>10. Does not assess impact of intervention(s) on patient and/or spouse/caregiver outcome(s) (e.g., study protocol with no results reported, description of development/adaptation of intervention, or evaluation of impact of intervention on other types of processes or outcomes)

Supplemental Table S4. Sample Demographics from the Studies Included in this Review.

Inter-vention	Citation	Study type	*N* dyads in final sample	Race/ ethnicity	Age (M, SD)	% Female	Annual Household Income (mode, unless otherwise indicated)	Education
4Cs program	Li et al. (2015)[62]	Single-arm trial	92	Not reported (study conducted in China with Mandarin speakers)	PT: 57.7, 11.4<br>SP: 56.8, 10.7	PT: 33.3%<br>SP: 66.7%	2001-6000 RMB/month (64.1% of sample)	*Mode:* "primary school or less"<br>PT: 50.4%<br>SP: 53.0%
ACT	Mosher et al. (2019)[63]	RCT: ACT vs. education/ support control condition	50	PT_{ACT}: 80.00% Non-Hispanic White<br>$PT_{control}$: 92.00% Non-Hispanic White<br>CG_{ACT}: 80.00% Non-Hispanic White<br>$CG_{control}$: 88.00% Non-Hispanic White	PT_{ACT}: 63.20, 11.27<br>$PT_{control}$: 62.00, 13.13<br>CG_{ACT}: 61.64, 11.52<br>$CG_{control}$: 52.40, 18.10	PT_{ACT}: 44.00%<br>$PT_{control}$: 44.00%<br>CG_{ACT}: 76.00%<br>$CG_{control}$: 84.00%	PT_{ACT}: $0-$50,999 (40%)<br>$PT_{control}$: $51,000-$99,999 (40%)<br>CG_{ACT}: $0-$50,999 (40%)<br>$CG_{control}$: $0-$50,999; $51,000-$99,999; $100,000 or more (32.00% each)	*Years (M, SD):*<br>PT_{ACT}: 14.28, 2.57<br>$PT_{control}$: 13.56, 2.65<br>CG_{ACT}: 14.44, 2.57<br>$CG_{control}$: 14.88, 2.71
CBM	Milbury et al. (2020)[37]	RCT: CBM vs. UC	35	PT_{CBM}: 78% Non-Hispanic White<br>$PT_{control}$: 81% Non-Hispanic White<br>SP_{CBM}: 72% Non-Hispanic White<br>$SP_{control}$: 75% Non-Hispanic White	PT_{CBM}: 57.67, 12.18<br>$PT_{control}$: 55.82, 11.00<br>SP_{CBM}: 56.67, 13.21<br>$SP_{control}$: 52.12, 10.95	PT_{CBM}: 44%<br>$PT_{control}$: 47%<br>SP_{CBM}: 66%<br>$SP_{control}$: 59%	≥$75,000<br>$PT_{CBM}$: 89%<br>$PT_{control}$: 71<br>$SP_{CBM}$: 89<br>$SP_{control}$: 76	*Mode:* "some college or more":<br>PT_{CBM}: 72%<br>$PT_{control}$: 59%<br>SP_{CBM}: 89%<br>$SP_{control}$: 71%
CDGI	Saita et al. (2016)[64]	RCT: CDGI vs. UC	50	Not reported (study conducted in Northern Italy with Italian speakers)	CDGI: 62, 8.80<br>Control: 62, 8.12	CDGI: 87.5%<br>Control: 79.4%	Not reported	*Mode:* "did not graduate from high school"<br>CDGI: 62.6%<br>Control: 58.8%
CECT	Collins et al. (2013)[65]	Single-arm pilot feasibility & acceptability trial	12	Not reported	*Median:* 64 (SD=8)	PT: 0%<br>SP: 100%	Not reported	*Mode:*<br>PT: "tertiary education" (70%)<br>SP: Not reported
	Couper et al. (2015)[66]	RCT: CECT vs. UC	62	Not reported	PT_{UC}: 65, 1.5<br>PT_{CECT}: 64, 1.9<br>SP_{UC}: 62, 1.5<br>SP_{CECT}: 60, 1.9	PT: 0%<br>SP: 100%	Not reported	*Mode:*<br>PT_{UC}: "Undergraduate" (28.13%)<br>PT_{CECT}: "High school certificate" (33.33%)<br>SP_{UC}: "Apprenticeship" (56.25%)<br>SP_{CECT}: "Apprenticeship" (59.38%)
COPE	McMillan et al. (2006)[52]	RCT: COPE + usual hospice care	329	Not reported	$PT_{COPE+UC}$: 70.84, 10.99<br>PT_{UC}: 70.12, 12.58<br>$PT_{UC+3\ visits}$: 71.02, 12.12	$PT_{COPE+UC}$: 36.9%<br>PT_{UC}: 44.0%<br>$PT_{UC+3\ visits}$: 38.5%	Not reported	*Years (M, SD):*<br>$PT_{COPE+UC}$: 11.84, 3.41<br>PT_{UC}: 12.49, 2.80

		(UC) vs. UC vs. UC + 3 supportive visits			$CG_{COPE+UC}$: 63.06, 13.58<br>CG_{UC}: 59.58, 15.27<br>$CG_{UC+3\ visits}$: 61.53, 15.47	$CG_{COPE+UC}$: 76.6%<br>CG_{UC}: 80.7%<br>$CG_{UC+3\ visits}$: 99.1 %		$PT_{UC+3\ visits}$: 12.28, 3.21<br>$CG_{COPE+UC}$: 13.05, 2.98<br>CG_{UC}: 12.86, 2.25<br>$CG_{UC+3\ visits}$: 12.70, 2.43
	McMillan & Small (2007)[32]	Secondary analysis of data from McMillan et al. (2006)[52]	329	See McMillan et al. (2006)[52]	See McMillan et al. (2006)[52]	See McMillan et al. (2006)[52]	See McMillan et al. (2006)[52]	See McMillan et al. (2006)[52]
	Meyers et al. (2011)[51]	RCT: COPE vs. UC	476	PT: 88% Caucasian/Other, 9% Hispanic (any race), 7% Asian/Pacific Islander, 5% African American, 1% Native American/Indigenous<br>CG: 85% Caucasian/Other, 8% Hispanic (any race), 8% Asian/Pacific Islander, 6% African American, 1% Native American/Indigenous	PT: 61.5 (SD not reported)<br>CG: 61.4 (SD not reported)	PT: 55%<br>CG: 31%	PT: >$80,000 (29%)<br>CG: Not reported	*Mode:* “College”<br>PT: 39%<br>CG: 47%
CSC	Gremore et al. (2021)[67]	Pilot RCT: CSC vs. UC	20	PT: 85% Caucasian, 15% Asian/Pacific Islander<br>SP: 80% Caucasian, 20% Asian/Pacific Islander	PT: 54, 15.5<br>SP: 53, 9.4	PT: 5%<br>SP: 95%	Not reported	*M years, SD:*<br>PT: 15, 4.1<br>SP: 15, 4.1
Dignity Therapy	Wang et al. (2021)[68]	RCT	68	Not reported (study conducted in China with Chinese speakers)	PT: 49.42, 12.33<br>CG: 38.30, 8.86	PT: 47.2%<br>CG: 45.3%	PT: 1000-3000 RMB/month (39.6%)<br>CG: Not reported	*Mode:* “Middle or high school”<br>PT: 58.5%<br>CG: 56.6%
DYP	Milbury, et al. (2018)[69]	Single-arm pilot study	5	PT: 60% Latino/Hispanic, 20% Non-Hispanic/White, 20% missing<br>CG: 80% Latino/Hispanic, 20% Non-Hispanic/White	PT: 51.94, 20.20<br>CG: 58.16, 10.15	PT: 80%<br>CG: 60%	≥$50,000<br>PT: 60%<br>CG: 80%	*Mode:* “Some college or higher”<br>PT: 80%<br>CG: 100%
	Millbury, Li, et al. (2019) [70]	Pilot RCT	20	PT_{DYP}: 90% Non-Hispanic/White, 10% Latino/Hispanic<br>$PT_{control}$: 80% Non-Hispanic/White, 20% Latino/Hispanic<br>CG_{DYP}: 80% Non-Hispanic/White, 10% Latino/Hispanic, 10% African American	PT_{DYP}: 47.91, 14.66<br>$PT_{control}$: 44.73, 12.23<br>CG_{DYP}: 52.36, 16.00<br>$CG_{control}$: 48.27, 11.88	PT_{DYP}: 50%<br>$PT_{control}$: 50%<br>CG_{DYP}: 70%<br>$CG_{control}$: 60%	≥$50,000 (100% of sample)	*Mode:* “Some college or higher”<br>PT_{DYP}: 100%<br>$PT_{control}$: 90%<br>CG_{DYP}: 80%<br>$CG_{control}$: 80%

				$CG_{control}$: 70% Non-Hispanic/White, 20% Latino/Hispanic, 10% >1 race				
	Millbury, Liao, et al. (2019)[71]	Pilot RCT	26	PT_{DYP}: 85% Non-Hispanic White<br>$PT_{control}$: 100% Non-Hispanic White<br>CG_{DYP}: 85% Non-Hispanic White<br>$CG_{control}$: 100% Non-Hispanic White	PT_{DYP}: 66.15, 5.48<br>$PT_{control}$: 65.54, 12.53<br>CG_{DYP}: 62.01, 11.37<br>$CG_{control}$: 56.9, 15.17	PT_{DYP}: 38%<br>$PT_{control}$: 38%<br>CG_{DYP}: 62%<br>$CG_{control}$: 83%	≥$50,000<br>$PT_{DYP}$: 92%<br>$PT_{control}$: 85%<br>$CG_{DYP}$: 85%<br>$CG_{control}$: 69%	*Mode:* "Some college or higher"<br>PT_{DYP}: 77%<br>$PT_{control}$: 77%<br>CG_{DYP}: 85%<br>$CG_{control}$: 69%
ECG	Manne et al. (2016)[53]	RCT: ECG vs. couples' support group (SG)	302	PT_{ECG}: 86.1% Non-Hispanic White, 9.3% Black, 2.6% Hispanic, 0.7% Asian, 0.7% Hawaiian/Pacific Islander, 0.7% Other<br>PT_{SG}: 85.4% Non-Hispanic White, 7.9% Black, 2.6% Hispanic, 1.3% Asian, 1.3% Other, 1.3% missing<br>CG_{ECG}: 80.1% Non-Hispanic White, 8.6% Black, 7.3% Hispanic, 2.0% Other, 1.3% missing, 0.7% Asian<br>CG_{SG}: 84.1% Non-Hispanic White, 8.6% Black, 2.6% Hispanic, 1.3% Asian, 1.3% Other, 1.3% missing	PT_{ECG}: 55.8, 10.9<br>PT_{SG}: 54.4, 9.9<br>SP_{ECG}: 57.1, 11.6<br>SP_{SG}: 55.5, 11.1	PT_{ECG}: 100%<br>PT_{SG}: 100%<br>SP_{ECG}: 1.4%<br>SP_{SG}: 0%	*Medians:*<br>PT_{ECG}: $95,000<br>$PT_{SG}$: $100,000<br>$SP_{ECG}$: $95,000<br>$SP_{SG}$: $100,000	*Mode:* "> college"<br>PT_{ECG}: 55.6%<br>PT_{SG}: 58.9%<br>SP_{ECG}: 54.3%<br>SP_{SG}: 60.0%
FOCUS	Northouse et al. (2002)[72]	RCT: FOCUS + UC vs. UC	117	Not reported	Not reported	PT: 100%<br>Family: Not reported	Not reported	Not reported
	Northouse et al. (2005)[73]	RCT: FOCUS + UC vs. UC	134	77% Caucasian, 19% African American, 4% Hispanic, Asian, or Native American	PT: 54, 11.0<br>CG: 52, 14.0	PT: 100%<br>CG: Not reported (between 13-35%)	*Median:* $30,000-$50,000	*Years (M, SD):*<br>14 (SD not reported)
	Northouse et al. (2007)[55]	RCT: FOCUS vs. UC	235	84% Caucasian, 14% African American, 2% Hispanic, Asian, Native American, or mixed race	PT: 63, 9.1<br>SP: 59, 9.7	PT: 0%<br>CG: Not reported	*Median:* $50,000-$75,000	*Years (M, SD):*<br>PT: 16 (SD not reported)<br>SP: 15 (SD not reported)
	Harden et al. (2009)[29]	Secondary analysis of data from Northouse et al. (2007)[55]	86	85% Caucasian	PT: 63.7 (SD not reported)<br>SP: 59.6 (SD not reported)	Not reported	≥$75,000 (54% of sample)	*Mode:* "had college degrees" (71% of sample)

	Northouse et al. (2013)[74]	RCT: brief FOCUS vs. extensive FOCUS vs. UC	302	82.5% Caucasian, 13.5% African American, 1.3% American Indian, 1.3% Asian, 1% Hispanic, 0.3% multi-racial	PT: 60.5, 10.9<br>CG: 56.7, 12.6	PT: 61.4%<br>CG: 55.8%	Not reported	*Years (M, SD):*<br>14.8, 2.7
	Northouse et al. (2014)[54]	Single-arm feasibility study	38	PT: 92.1% White, 5.3% African American, 2.6% Asian<br>CG: 92.1% White, 5.3% African American, 2.6% Asian	PT: 54.8, 12.6<br>CG: 50.6, 14.7	PT: 57.9%<br>CG: 60.5%	$50,001-$100,000<br>PT: 44.7%<br>CG: 36.8%	*Mode:* "postcollege"<br>PT: 34.2%<br>CG: 42.1%
	Martinez et al. (2015)[31]	Secondary analysis of data from Northouse et al. (2013)[74]	484 patients	78.9% White, 14.5% Black, 6.6% Other	60.5 (SD not reported)	62%	$30,000-$74,999 (34% of sample)	*Mode:* "some college or more" (65% of sample)
	Dockham et al. (2016)[75]	Single-arm pilot effectiveness study	34	PT: 89.2% White, 5.4% African American, 2.7% Asian, 2.7% Native American<br>CG: 86.5% White, 5.4% African American, 2.7% Asian, 2.7% Native American, 2.7% missing	PT: 53.8, 11.0<br>CG: 53.4, 11.1	PT: 73.0%<br>CG: 35.1%	PT: $75,001-$100,000 and >$100,000 (18.9% each)<br>CG: $50,001-$75,000 (27.0%)	*Mode:*<br>PT: "Graduate [school]" (40.5%)<br>CG: "Bachelor's degree" (43.2%)
	Titler et al. (2017)[56]	Single-arm trial	36	PT: 88.9% White, 2.8% Asian, 2.8% Black/African American, 2.8% Latino/Hispanic, 2.8% Other<br>CG: 77.8% White, 8.3% Asian, 2.8% Black/African American, 2.8% Latino/Hispanic, 2.8% Other, 5.5% missing	PT: 60.8, 14.2<br>CG: 55.9, 15.1	PT: 52.8%<br>CG: 55.6%	≥$100,000<br>PT: 44.4%<br>CG: 41.7%	*Mode:*<br>PT: "Graduate degree" (47.2%)<br>CG: College graduate (38.9%)
	Chen et al. (2021)[76]	Single-arm pilot study	29	90.0% White, 3.3% Asian, 3.3% Black, 3.3% Latino/Hispanic	PT: 68, 11<br>CG: 61.7, 13.5	PT: 70.0%<br>CG: 56.7%	Not reported	*Mode:*<br>PT: "High school" (33.3%)<br>CG: "College or some college" (50.0%)
	Titler et al. (2020)[77]	Secondary analysis of data from Titler et al. (2017)[56]	36	See Titler et al. (2017)[56]	See Titler et al. (2017)[56]	See Titler et al. (2017)[56]	See Titler et al. (2017)[56]	See Titler et al. (2017)[56]
I-BMS	Lau et al. (2020)[35]	RCT: I-BMS vs. CBT (same data as Xiu et al. (2020);[36]	157	Not reported (study conducted in Hong Kong with Chinese patients & their caregivers)	PT: 60.0, 9.5<br>CG: 53.9, 12.2	PT: 59.9%<br>CG: 52.2%	Not reported	*Mode:* "secondary"<br>PT: 47.1%<br>CG: 46.5%

		report on PT outcomes)						
	Xiu et al. (2020)[36]	RCT: I-BMS vs. CBT (same data as Lau et al. (2020);[35] report on CG outcomes)	157	See Lau et al. (2020)[35]	See Lau et al. (2020)[35]	See Lau et al. (2020)[35]	See Lau et al. (2020)[35]	See Lau et al. (2020)[35]
MBSR	Birnie et al. (2010)[78]	Single-arm trial	21	Not reported (study conducted in Canada)	PT: 62.9, 7.4<br>SP: 62.8, 9.3	PT: 52.4%<br>SP: 47.6%	Not reported	*Years (M, SD):*<br>PT: 14.9, 3.6<br>SP: 14.3, 4.2
MBSR-C	Lengacher et al. (2012)[80]	Single-arm pilot study	26	PT: 73.1% White Non-Hispanic, 15.4% White Hispanic, 11.5% Black Non-Hispanic<br>CG: 88.5% White Non-Hispanic, 3.8 White Hispanic, 3.8% Black Non-Hispanic, 3.8% Black Hispanic	PT: 53.5, 10.4<br>CG: 51.5, 14.6	PT: 69.2%<br>CG: 61.5%	Not reported	*Mode:* "some college"<br>PT: 38.5%<br>CG: 38.5%
MODEL Care	Cottingham et al. (2019)[81]	Secondary analysis of Johns et al. (2020)[79]	12	See Johns et al. (2020)[79]	See Johns et al. (2020)[79]	See Johns et al. (2020)[79]	See Johns et al. (2020)[79]	See Johns et al. (2020)[79]
	Johns et al. (2020)[79]	Single-arm pilot study	13	PT: 92.31% White/Caucasian, 7.69% Black/African American<br>CG: 69.23% White/Caucasian, 15.4% Black/African American, 7.69% American Indian/Alaska Native, 7.69% Asian	PT: 62.9, 10.6<br>CG: 56.6, 15.6	PT: 46.2%<br>CG: 76.9%	"Comfortable"<br>PT: 69.2%<br>CG: 61.5%	*Mode:* At least "Some college" PT: 76.9%<br>CG: 84.6%
PIP/MPI	Chien et al. (2020)[82]	RCT: PIP vs. MPI vs. control	103	100% Asian	PT: 67.8, 6.2<br>SP: 63.6, 7.3	PT: 0%<br>SP: Not reported	Not reported	*Mode:*<br>PT: "college or above" (45.6%)<br>SP: "high school" (41.7%)
PERC	Song et al. (2015)[61]	Single-arm pilot feasibility & acceptability study	22	PT: 72.7% White, 27.3% Black<br>SP: Not reported	PT: 62.9, 8.2<br>SP: 59.3, 10.7	PT: 0%<br>SP: Not reported	>$50,000 (68.2% of sample)	*Mode:* College degree or higher<br>PT: 68.2%<br>SP: 72.7%
PICP	Kayser et al. (2010)[83]	RCT: PICP vs. hospital standard	47	90% White	PT: 46.3, 9.8<br>SP: 48.7, 10.7	Not reported	>$90,000 (69.6% of sample)	*Mode:* College graduate<br>PT: 87.3%<br>SP: 89.4%

		social work services						
PSE	Bevans et al. (2010)[84]	Single-arm pilot feasibility study	8	PT: 87.5% White Non-Hispanic, 12.5% White Hispanic<br>SP: 87.5% White Non-Hispanic, 12.5% White Hispanic	PT: 56.5, 7.9<br>SP: 53.9, 9.7	PT: 25.0%<br>SP: 62.5%<br>*[Note: The text states, "All dyads were husband and wife pairs," but Table 1 (demographics) reports that 75.0% of patients and 37.5% of caregivers were male.]*	Not reported	*Mode:* "At least some college"<br>PT: 75%<br>SP: 75%
RE	Baucom et al. (2009)[85]	Pilot RCT: RE vs. UC	14	86% White	*Median:* 50	PT: 100%<br>SP: 0%	Not reported	*Median:* 16 years
RIPSToP	Robertson et al. (2016)[86]	RCT: RIPSToP vs. UC	43	100% White	PT: 63.7<br>SP: Not reported	PT: 0.0%<br>SP: 95.5%	Not reported	Not reported
Side by Side	Heinrichs et al. (2012)[87]	RCT: Side by Side vs. control	72	Not reported	PT: 52.2, 11.3<br>SP: 52.7, 11.4	PT: 100%<br>SP: 0%	Not reported	*Mode:* "more than 10 years"<br>PT: 44%<br>SP: 54%
TSM	Mosher et al. (2016)[50]	RCT: TSM vs. education control condition	106	PT: 90.6% Non-Hispanic White<br>CG: 89.6% Non-Hispanic White, 0.9% missing	PT: 62.7, 7.9<br>CG: 56.5, 13.9	PT: 52.9%<br>CG: 89.5%	$21,000-$50,999<br>PT: 30.8%<br>CG: 34.5%	*Years (M, SD):*<br>PT: 13.1, 2.1<br>CG: 13.6, 2.6
	Winger et al. (2018)[34]	Secondary analysis of data from Mosher et al. (2016)[50]	51	PT: 88.2% Non-Hispanic White<br>CG: 86.3% Non-Hispanic White, 1.9% missing	PT: 63.5, 7.7<br>CG: 56.3, 14.1	PT: 54.9%<br>CG: 72.5%	$51,000-$99,000<br>PT: 25.5%<br>CG: 33.3%	*Years (M, SD):*<br>PT: 12.9, 2.2<br>CG: 13.9, 2.8
TYC	Milbury et al. (2015)[88]	Single-arm pilot study	10	PT: 80.0% White, 10.0% Latino/Hispanic, 10.0% Other<br>CG: 80.0% White, 10.0% Latino/Hispanic, 10.0% Other	PT: 71.22, 6.16<br>CG: 68.77, 5.99	PT: 35.7%<br>CG: 64.3%	≥$50,000<br>PT: 70.0%<br>CG: 80.0%	*Mode:* "Some college or higher"<br>PT: 50.0%<br>CG: 57.1%
Unnamed	Shields et al. (2004)[89]	Non-randomized, controlled trial: 2-session intervention vs. 1-session intervention vs. control	48	Not reported	PT: 55.7, 11.7<br>SP: 60.6, 9.7	PT: 100%<br>SP: 0%	*M, SD:*<br>PT: $70,553, $28,871<br>SP: $72,840, $28,962	*Years (M, SD):*<br>PT: 15.7, 2.5<br>SP: 15.6, 2.5
Unnamed	Wagner et al. (2016)[90]	Single-arm pilot study	12	Not reported	PT: 59.1<br>SP: 59.6	PT: 63.6%<br>SP: 54.5%	$70,000<br>PT: Not reported	*Mode:* High School<br>PT: 45.4%

							SP: 54.5%	SP: 54.5%
Unnamed	Mosher et al. (2018)[60]	RCT: peer helping + coping skills vs. coping skills	50	PT: 86% Non-Hispanic White<br>CG: 88% Non-Hispanic White	PT: 58.2, 11.5<br>CG: 53.8, 13.6	PT: 38%<br>CG: 66%	$51,000-$99,999<br>PT: 38%<br>CG: 40%	*Years (M, SD):*<br>PT: 14.6, 2.6<br>CG: 14.6, 2.1
Unnamed	Clark et al. (2013)[91]	RCT: intervention vs. UC	117	PT: 97% White<br>CG: Not reported	PT: 59.3, 10.7<br>CG: Not reported	PT: 34.5%<br>CG: Not reported	Not reported	Not reported
	Piderman et al. (2014)[33]	Secondary analysis of data from Clark et al. (2013)[91]	117	See Clark et al. (2013)[91]	See Clark et al. (2013)[91]	See Clark et al. (2013)[91]	See Clark et al. (2013)[91]	See Clark et al. (2013)[91]
	Lapid et al. (2016)[30]	Secondary analysis of data from Clark et al. (2013)[91]	116	See Clark et al. (2013)[91]	See Clark et al. (2013)[91]	See Clark et al. (2013)[91]	See Clark et al. (2013)[91]	See Clark et al. (2013)[91]
